# Testing an infection model to explain excess risk of preterm birth with long-term iron supplementation in a malaria endemic area

**DOI:** 10.1186/s12936-019-3013-6

**Published:** 2019-11-26

**Authors:** Bernard Brabin, Halidou Tinto, Stephen A. Roberts

**Affiliations:** 1 Department of Clinical Sciences, Liverpool School of Tropical Medicine, Liverpool, L35QA UK; 2 Institute of Infection and Global Health, University of Liverpool, Liverpool, UK; 3 Global Child Health Group, Academic Medical Centre, University of Amsterdam, Amsterdam, The Netherlands; 4 Clinical Research Unit of Nanoro (URCN/IRSS), Nanoro, Burkina Faso; 5 Centre for Biostatistics, Division of Population Health, Health Services Research and Primary Care, Faculty of Biology, Medicine and Health, University of Manchester, Manchester Academic Health Science Centre (MAHSC), Manchester, UK

**Keywords:** Dual infection model, Iron, Inflammation, Hepcidin, Preterm birth, Malaria

## Abstract

**Background:**

In view of recent evidence from a randomized trial in Burkina Faso that periconceptional iron supplementation substantially increases risk of spontaneous preterm birth (< 37 weeks) in first pregnancies (adjusted relative risk = 2.22; 95% CI 1.39–3.61), explanation is required to understand potential mechanisms, including progesterone mediated responses, linking long-term iron supplementation, malaria and gestational age.

**Methods:**

The analysis developed a model based on a dual hit inflammatory mechanism arising from simultaneous malaria and gut infections, supported in part by published trial results. This model is developed to understand mechanisms linking iron supplementation, malaria and gestational age. Background literature substantiates synergistic inflammatory effects of these infections where trial data is unavailable. A path modelling exercise assessed direct and indirect paths influencing preterm birth and gestation length.

**Results:**

A dual hit hypothesis incorporates two main pathways for pro-inflammatory mechanisms, which in this model, interact to increase hepcidin expression. Trial data showed preterm birth was positively associated with C-reactive protein (P = 0.0038) an inflammatory biomarker. The malaria pathway upregulates C-reactive protein and serum hepcidin, thereby reducing iron absorption. The enteric pathway results from unabsorbed gut iron, which induces microbiome changes and pathogenic gut infections, initiating pro-inflammatory events with lipopolysaccharide expression. Data from the trial suggest that raised hepcidin concentration is a mediating catalyst, being inversely associated with shorter gestational age at delivery (P = 0.002) and positively with preterm incidence (P = 0.007). A segmented regression model identified a change-point consisting of two segments before and after a sharp rise in hepcidin concentration. This showed a post change hepcidin elevation in women with increasing C-reactive protein values in late gestation (post-change slope 0.55. 95% CI 0.39–0.92, P < 0.001). Path modelling confirmed seasonal malaria effects on preterm birth, with mediation through C-reactive protein and (non-linear) hepcidin induction.

**Conclusions:**

Following long-term iron supplementation, dual inflammatory pathways that mediate hepcidin expression and culminate in progesterone withdrawal may account for the reduction in gestational age observed in first pregnancies in this area of high malaria exposure. If correct, this model strongly suggests that in such areas, effective infection control is required prior to iron supplementation to avoid increasing preterm births.

*Trial registration* NCT01210040. Registered with Clinicaltrials.gov on 27th September 2010

## Background

In 2014, estimates for prevalence of PTB in sub-Saharan Africa based on a variety of assessment methods ranged from 8.6 to 16.7% [1]. PTB prevalence and mean gestational age estimates for sub-Saharan African countries dated by ultrasound, which is the most accurate measurement are shown in Table 1. These estimates are compared with those for high income, non-malaria endemic countries. Highest PTB prevalence (27.5%) was observed for women receiving long-term and periconceptional iron in a randomized double blind controlled safety trial of iron supplementation before the first pregnancy in Burkina Faso (PALUFER) [2]. All women in the trial were young primigravidae and in a Malian study, under similar malaria endemic conditions to those in Burkina Faso, comparable gestational age results were observed in first pregnancies. Mean ultrasound dated gestational age was 268.9 days and PTB prevalence 15.8% in Mali [3], very similar to the 269 days and 13.9% prevalence recorded for the control arm in the PALUFER trial (Table 1). In both studies, all women received routine antenatal care, including daily iron prescription in pregnancy. Comparison with other studies listed in Table 1 is limited because none report outcomes separately for primigravidae. There is a need to substantiate the basis for this high PTB prevalence in primigravidae in Burkina Faso and the potential influence of long-term iron supplementation.

**Table 1:** Ultrasound-dated preterm birth prevalence and mean gestational age in malaria endemic and non-endemic areas

Country	Study years	Study design^a^	Age^b^	N	Parity	Mean gestational age (days)	Preterm birth^c^ (%)	Malaria exposure	References
Malawi	Pre 2009	PRCT	≤ 20–≥ 40	2149	All	270.2	16.3	29.5^d^	[92]
	Pre 2005	Cohort	22.8	456	All	266	20.3	NR	[97]
Mali	2010–2013	Cohort	15–45	152	PG	268.8	16.4	15.8^e^	[3]
	–		–	114	SG	271.5	12.3	10.5^e^	
	–		–	325	MG	275.8	2.5	9.5^e^	
Kenya									
Control	2011–2013	PRCT	15–45	233	All	271	16.2	52.1^f^	[93]
Iron supplement	–			237	All	274.4	9.1	50.9	
Gambia									
Control	2006–2008	Peri-RCT	28.9	150	All	282.1	5.3	1.3^g^	[98]
Multinutrients			–	139	All	280	0.7	0.9	
Benin	2008–2011	Cohort	NR	814	All	NR	9.9	34.2^h^	[84]
Tanzania	2008–2010	Cohort	22	28	PG,SG	272.7	NR	All exposed^i^	[83]
	–	–	23	93	PG,SG	279.4	NR	None exposed^i^	
Uganda	2014	Nested PRCT	22.1	282	All	NR	9.2	2.9 to 8.6^j^	[94]
Papua New Guinea	2009–2012	PRCT	24.5	1941	All	274	8.6	13.6^k^	[95]
Burkina Faso (PALUFER)									
Control	2011–2013	Peri-RCT	17.1	137	PG	269	13.9	29.3^l^	[2]
Iron supplemented	–	–	–	149	PG	264	27.5	37.1^l^	
34 high-income countries	1996–2010	Mixed	NR	9.1 × 10^6^	All	275.5	4.6–8.2	None	[96]

*PG* primigravidae, *SG* secundigravidae; multigravidae, *N* sample size, *NR* Not reported, *PRCT* pregnancy randomized control trial, *Peri-RCT* periconceptional randomized control trial

^a^Provision of antenatal iron supplements stated for Malawi, Kenya (intervention arm alone), Gambia, Burkina Faso (both trial arms after first antenatal visit). Not stated for other studies or malaria non-endemic areas

^b^Mean, median, or range in years

^c^Birth less than 37 weeks

^d^Antenatal parasite prevalence from thick blood film on peripheral blood at booking and second antenatal visit

^e^Parasite prevalence in peripheral blood or placental blood smear

^f^Prevalence estimate from at least one positive result for dipstick tests (HRP2 or pLDH for any *Plasmodium* species) in maternal venous or placental blood, or by *P. falciparum*—specific PCR tests in maternal erythrocytes from venous or placental blood, or presence of parasites or pigment in placental biopsies by histopathology. Malaria estimates include past infections in pregnancy

^g^Malaria parasitaemia prevalence at enrolment in placebo cohort; sample size n = 240 (control), n = 232 (micronutrients)

^h^Sub-microscopic prevalence at delivery on capillary venous blood with Real-time PCR Assay for detection of *P. falciparum* infections

^i^Exposed or not exposed to malaria early in pregnancy

^j^Prevalence by microscopy of placental blood smear (2.9%); prevalence of parasite DNA in placental blood (8.6%), and histopathologic detection of malaria infection (pigment or parasites) of placental biopsies, which includes past infection (37.2%)

^k^Peripheral parasitaemia prevalence at enrolment by light microscopy and/or real time polymerase chain reaction (*P. falciparum*, *P. vivax*)

^l^Acute and chronic placental malaria prevalence (presence of parasitized cells on histology). Sample size at delivery n = 89 (iron supplements, n = 92 (control))

Hepcidin is an iron-regulatory hormone produced in the liver that controls the entry of iron into the circulation and tissue iron distribution [4]. Hepcidin exerts its iron-regulatory effects by binding to the transmembrane iron exporter, ferroportin, causing cellular ferroportin internalization and degradation. Thus, increased hepcidin concentration inhibits duodenal iron absorption where ferroportin is needed to deliver absorbed dietary iron to the circulation [4]. Elevated hepcidin thus decreases dietary enteric iron absorption while increasing iron availability to bacterial and fungal pathogens that thrive on gut iron. Iron supplementation would potentially affect iron homeostasis and hepcidin expression if unabsorbed gut iron increased gut inflammation which could stimulate hepcidin production.

In the PALUFER trial malaria prevalence was high above 50% [5], but as most were chronically asymptomatic adolescents, failure to treat these infections probably led to inflammation and gut tissue pathology including detachment of epithelia and shortening of colonic villi as a consequence off epithelial parasite sequestration [6]. The trial documented increased administration of antibiotics for enteric infections as well as antifungal prescriptions for genital infections in supplemented women [7]. As serum iron biomarkers were not improved with supplementation, impaired iron absorption was inferred [5] and serum hepcidin implicated as a key mechanism modulating malarial and gut infections. Preterm birth (PTB) incidence during the rainy season was two and half times higher in the iron arm (P = 0.001) [2], and it was suggested that inflammation related to gut infection and seasonal malaria were initiating this increase. Elevated serum hepcidin and C-reactive protein concentrations were present in malaria parasitaemic, compared to non-parasitaemic, women in the trial [5, 8]. The enteric pathway is developed in the model presented as substantial published information is available to assess its potential influence on host inflammation following long-term iron supplementation. As *Plasmodium falciparum* parasites sequester in gut epithelium secondary effects on intestinal cell integrity, cell signalling and permeability are considered.

The PALUFER trial has previously been described in detail (see references in “Methods”). Briefly two cohorts of supplemented women were followed: women remaining non-pregnant and those who experienced pregnancy during the 18-month iron supplementation period. Nulliparous participants were individually randomized to receive either a weekly capsule containing ferrous gluconate (60 mg) and folic acid (2.8 mg) (n = 980), or an identical capsule containing folic acid alone (2.8 mg) (n = 979) for 18 months, or until attendance at a first study antenatal visit (ANC1). A total of 478 women became pregnant. Median weekly supplement adherence was 79%. A total of 979 women remained non-pregnant and these were assessed for secondary outcomes after 18 months weekly supplementation. The primary study end-point was malaria parasitaemia prevalence at ANC1; the secondary end-points were prevalence of anaemia and iron deficiency at ANC1, and incidence of low birthweight and PTB. Weekly iron did not significantly reduce iron deficiency, or anaemia prevalence at ANC1. *Plasmodium* parasitaemia prevalence by microscopy was 54.3%, at ANC1, and prevalence did not differ by trial arm. Free treatment was available for women with fever or other malaria symptoms, but most trial participants were asymptomatic (6.7% with malaria and fever at ANC1). Prevalence of placental malaria parasites at delivery was 33%. In women remaining non-pregnant parasitaemia prevalence was 41% at end assessment after 18 months weekly iron supplementation. Iron-supplemented non-pregnant women also received more antibiotic treatments for non-genital infections (P = 0.014); mainly gastrointestinal infections (P = 0.005), anti-fungal treatments for genital infections (P = 0.014) and analgesics (P = 0.008), than controls.

On the basis of experimental research in animals, Romero et al. in 2014 proposed a two-hit infection hypothesis to account for spontaneous PTB [9, 10], noting the case in humans remained to be established. The PALUFER trial may be an example in humans of enteric iron induced excess preterm incidence due to such a dual hit inflammatory mechanism. In this paper, a model is formulated for malaria and enteric dual infections in order to investigate their synergistic relationship in the presence of long-term periconceptional iron supplementation. To test the hypothesis PALUFER trial data were used to model the dual pathways of malaria and gut infection that predispose to preterm birth and gestation. Data available from the trial can only substantiate part of the model, but data from other studies were used to complete the description of potential pathways for the observed effect of long-term iron supplementation on increased PTB risk. The model tested is that malarial infection induces an elevated hepcidin response that blocks iron absorption, causing gut infections that promote inflammatory pathways leading to PTB. Iron homeostasis is tightly regulated by the membrane iron exporter ferroportin and its regulatory peptide hormone hepcidin. This simple model is in reality more complex, not least because improving iron status (which did not occur in the PALUFER trial) probably also increases the risk of infection as malaria parasites and many bacteria are dependent on iron availability for their growth and virulence [11].

Improved understanding of such interactions may help formulate novel strategies for reducing PTB in malaria endemic regions. This is especially relevant as the World Health Organization currently recommends iron supplementation of 30–60 mg/day iron for 3 consecutive months in a year for non-pregnant females of reproductive age (menstruating adult women and adolescent girls) to better prepare girls for their first pregnancy [12]. Whether this recommended dose is safe or even effective in malaria-endemic settings is not clear.

## Methods

### Search strategy and selection criteria

Background and published data on the PALUFER trial, its design and results [2, 5, 7, 8, 13–15], are summarized in Additional file 1. Laboratory methods are outlined in Additional file 2.

Literature was searched for references illustrative of plausible mechanisms operating for each stage of the model pathways leading to PTB in a malaria endemic area. Search terms focussed on markers of inflammation that could affect birth outcomes. Assumptions for an inflammation-driven model for iron induced excess spontaneous preterm births in the PALUFER trial were formulated derived primarily from published trial results.

### Statistical methods

Relationships between PTB and biomarkers were assessed using logistic regression models, plotting fitted probabilities with associated 95% CI. The relationship between maternal serum hepcidin and CRP concentrations was strongly non-linear and was fitted using a segmented linear regression model which fits a continuous line composed of straight-line segments with a change of slope at a fitted change point. More complex models were considered including trial arm as a covariate, but as there were no substantive effects of supplementation, these were not more informative and the univariate analyses are presented. Biomarker levels between PTB and term birth groups were compared using Mann–Whitney U-tests. A path model was constructed based on the formulated schematic which incorporated C-reactive protein (CRP) and hepcidin mediation pathways and PTB. Season was used a proxy for malaria infection. The model was fitted to the PALUFER data as described in Additional file 4.

## Results

### Assumptions for an inflammation-driven model for iron induced excess spontaneous preterm births in the PALUFER trial

**Fig. 1:**
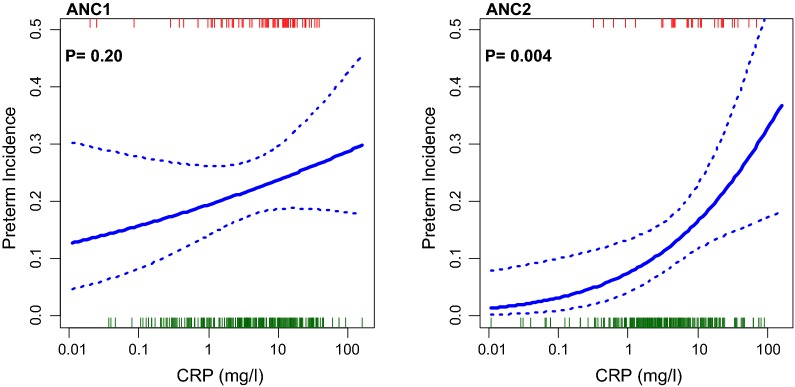
Association between maternal serum C-reactive protein concentration and spontaneous preterm birth in the PALUFER study. Fit and P-values derived from a logistic regression of preterm birth incidence (proportion) against log(CRP mg/l). Stippled lines are 95% confidence interval. Rugs at top and bottom indicate where preterm birth (red) and non-preterm birth (green) deliveries lie. ANC1 and ANC2 are the scheduled study antenatal visits. The regression slopes are 0.11 (95% CI − 0.06:0.29) for ANC1 and 0.39 (0.11:0.67) for ANC2 per unit log(CRP)

**Fig. 2:**
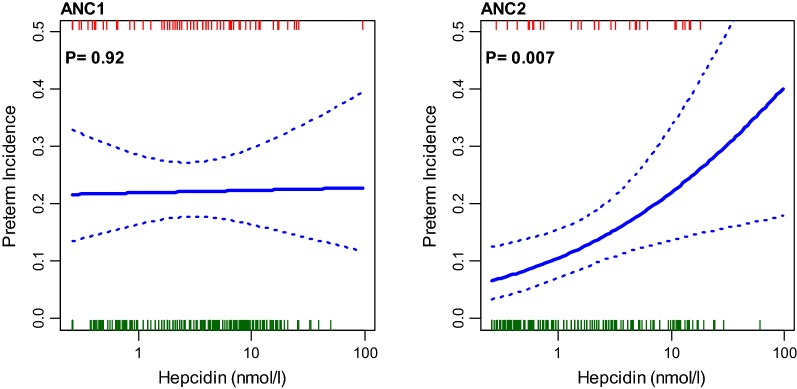
Association between maternal serum hepcidin concentration and spontaneous preterm birth in the PALUFER study. Fit and P-values derived from a logistic regression of preterm birth incidence (proportion) against log(hepcidin nmol/ml). Stippled lines are 95% confidence interval. Rugs at top and bottom indicate where preterm birth (red) and non-preterm birth (green) deliveries lie. ANC1 and ANC2 are the scheduled study antenatal visits. The regression slopes are 0.01 (95% CI − 0.20:0.22) for ANC1 and 0.38 (0.10:0.66) for ANC2 per unit log(hepcidin)

The trial results showed associations between maternal serum CRP concentrations > 5 mg/l at the first (median gestation 18 weeks, IQR 14–23 weeks) and second (median gestation 34 weeks, IQR 33–35 weeks) scheduled antenatal visits (ANC1, n = 282; ANC2, n = 239) and spontaneous PTB incidence (< 37 weeks gestation) [2]. The adjusted relative risk for PTB was higher with CRP > 5 mg/l at ANC1 (1.60, 95% CI 1.00–2.5, P = 0.04) and at ANC2 2.06 (1.04–4.10, P = 0.034). Figure 1 shows the linear association with 95% confidence interval of PTB incidence by log (CRP) for these two visits. There was a strong positive trend with CRP at ANC2 (P = 0.0038), suggesting this inflammatory response was related to spontaneous PTB. Furthermore, the whole gestational age distribution curve was shifted to the left in the iron-supplemented women (see Additional file 3), indicating a population effect on shortening gestation, thereby increasing PTB incidence [2]. Malaria associated inflammation as represented by CRP (geometric mean CRP, mg/l, (n) 1.3 [95% CI 1.0:1.7] (142) without parasitaemia; 9.2 [7.8:10.9] (169) with parasitaemia; relative risk: 1.97 [1.66:2.29], P < 0.001) upregulates hepcidin [8, 16]. At ANC2 (but not ANC1) linear regression showed maternal serum hepcidin concentration was inversely associated with gestational age at delivery (P = 0.002). The linear association and 95% confidence interval of log hepcidin by PTB incidence for these two visits is shown in Fig. 2 with significance only at ANC2 (P = 0.007). Mean ± SD serum hepcidin concentration was higher at both ANC1 and ANC2 in preterm compared to term deliveries (ANC1, 6.9 ± 13.5 vs 5.7 ± 6.9 nmol/l, P = 0.74; ANC2, 4.8 ± 5.3 vs 3.2 ± 6.3 nmol/l, P = 0.004).

**Fig. 3:**
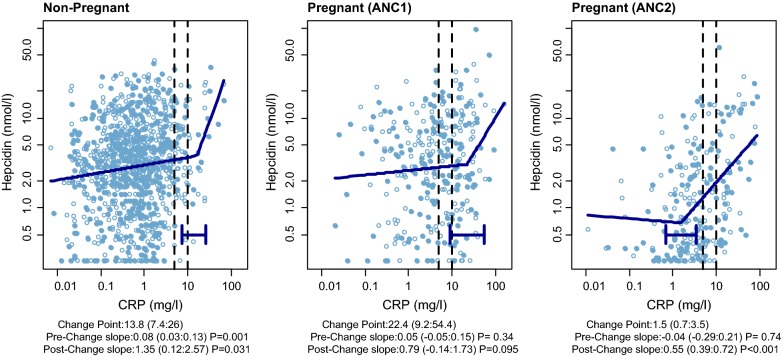
Association of maternal serum hepcidin and C-reactive protein concentration at the three assessment time-points in the PALUFER study. The lines show a segmented regression model consisting of linear segments before and after a (fitted) change point, and the different regression slopes before and after this change point with associated 95% CI are shown below the plots. The 95% confidence interval is shown for the change point estimate as a horizontal error bar and numerically below the plot. The vertical stippled lines show the cut points for CRP at 5 mg/l and 10 mg/l. Open and closed symbols indicate iron intervention (closed) and control (open) arms. Serum hepcidin is in nmol/l

In normal pregnancy hepcidin expression decreases physiologically in the second and third trimesters, thereby increasing the supply of iron to the circulation [17]. As malaria risk also alters with gestational age, especially in primigravidae, gestational change in hepcidin may be inhibited by concurrent malaria inflammation. Figure 3 shows plots of the relationship between maternal serum hepcidin and CRP concentrations at the three trial assessment time-points: end assessment for women remaining non-pregnant; for pregnant women at ANC1 and at ANC2. The figure shows a segmented regression model consisting of two segments before and after a sharp rise (post-change) in hepcidin concentration. In the PALUFER trial there was a post-change hepcidin elevation at ANC2 (post-change slope 0.55; 95% CI 0.39–0.92; P < 0.001).

### Iron induced enteric inflammation before and during pregnancy

**Fig. 4:**
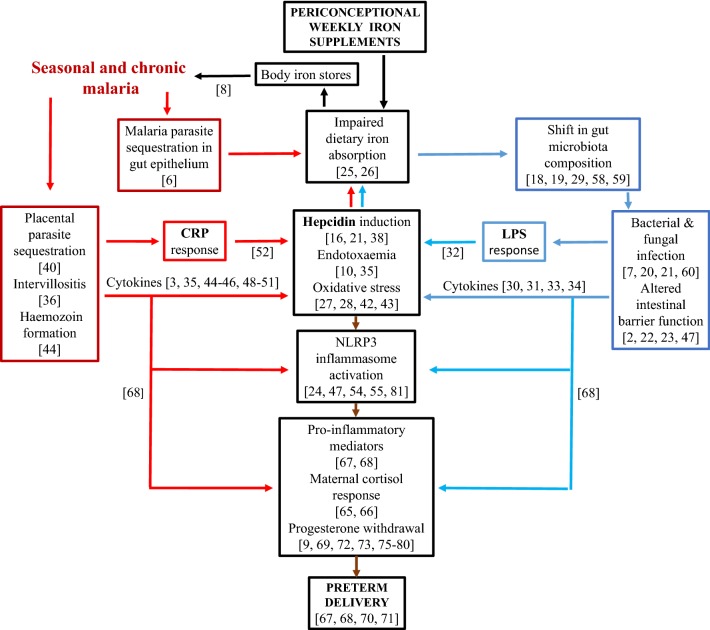
Synergistic effects of dual exposure with chronic malaria and enteric infection in iron supplemented adolescents and increased risk of preterm birth. Red arrows: malaria loop; blue arrows: enteric loop; black arrows: iron pathway; brown arrows: preterm pathway. Numbers in square brackets refer to manuscript references with evidence for the specific pathway events. Box texts refer to pathophysiological consequences and stages in the specific pathways. Body iron stores refers to the observation that in the PALUFER trial mean body iron stores were higher in pregnant women with malaria [14], indicating that better iron status was associated with increased malaria infection risk. Nulliparous participants were individually randomized to receive either a weekly capsule containing ferrous gluconate (60 mg elemental iron, 479 mg gluconate) and folic acid (2.8 mg), or an identical capsule containing folic acid alone (2.8 mg). CRP: C-reactive protein; NO: nitric oxide; LPS: lipopolysaccharide; pro-inflammatory cytokines are interleukin (IL)-1 beta (β); IL-6; IL-8; interferon (IFN) gamma (ɣ); Nod-Like Receptor (NLR)P3: gene belonging to the NLRP3 inflammasome complex

Pathological changes (including detachment of epithelia and shortening of colonic villi) occur in the permeability, leakage of infected erythrocytes into the lumen and dysbiosis of the intestinal gut during *P. falciparum* infection. Such changes may cause increased intestinal microbiota [6]. The extra enteric iron available with long-term supplementation potentially contributes to disease severity in the gut with a shift in microbiota composition [18, 19] and multiplication of fungal [20] and bacterial [21] pathogens that produce intestinal epithelium biofilm and also contaminate the genital tract. Expression of regulatory inflammatory genes can cause intestinal inflammation, disrupt the intestinal barrier function [22, 23] and prime inflammasome activation. The latter is a multiprotein oligomer responsible for the activation and assembly of the Nod-Like Receptor (NLR)P3 leading to release of pro-inflammatory cytokines, including IL-β [24]. Malaria infection decreases absorption of fortification iron in women [25], which can induce an inflammatory gut reaction [26, 27]. In this way, *Plasmodium* parasites can have an impact on composition of gut microbiota although bi-directional mechanisms are not well understood, especially in pregnancy [6]. Reactive nitric oxide (NO) intermediates play a prominent role in intestinal barrier damage by inducing enterocyte apoptosis and inhibiting the epithelial restitution processes [28, 29]. NO generation, produced by inducible NO synthase (iNOS), occurs with asymptomatic malaria. Some of the changes related to inflammation from gut infection and which activate pro-inflammatory factors are outlined in Fig. 4. This shows a positive feedback loop with hepcidin expression resulting from gut infection limiting dietary iron absorption, with enteric iron enhancing gut inflammation. Long-term iron supplementation would be expected to exacerbate this cycle as this in addition to food iron increases enteric iron concentration, especially in iron replete women whose iron absorption is already inhibited.

Figure 4 highlights the importance of release of endotoxins and lipopolysaccharide (LPS) into the circulation with inflammation [30, 31]. LPS is a central component of the outer membrane in Gram-negative bacteria and frequently plays a key role during host–pathogen interaction and the establishment of chronic infection in the gut, genital tract and other mucosa. LPS-mediated virulence resides in its endotoxic activity [32]. LPS induces release of acute phase blood proteins associated with pro-inflammatory signalling pathways. Studies in experimental animals have shown intestinal bacteria, especially Enterobacteriaceae and *Clostridium perfringens*, may influence the level of lipopolysaccharide binding protein and CRP in blood plasma [33] Strong activation is reported in macrophages in response to LPS of NF-κB, a highly conserved transcription factor that regulates inflammatory responses, cellular growth and apoptosis [34]. Placental mediated responses to LPS have been reported in experimental animals with greater cytokine responses in preterm placentas and increased phosphorylated NF-κB induction which may occur by activation of the Toll-like receptor (TLR) pathway [35]. TLRs are essential for an effective host cell response to LPS. Circulating LPS binding to membrane-bound TLR4 results in a series of kinase cascades that phosphorylate NFκB into the nucleus where it acts as a transcription factor stimulating production of proinflammatory cytokines. Placental TLRs can result from transient clinically inapparent maternal bacteraemia [36].

Hepcidin expression is reported to negatively affect proliferation of intrahepatic sporozoites [37], but conversely it may increase susceptibility to iron dependent gut pathogens, e.g., *Salmonella*. This is because iron-loaded macrophages, which increase with hepcidin expression, have an impaired potential to kill various pathogens. This is partly attributed to reduced formation of NO which is essential to fight infection, as iron blocks transcription of inducible NO synthase (iNOS) [38]. Regulatory mechanisms would depend on the intracellular or extracellular location of bacteria.

### Placental malaria and malaria-induced gut inflammation

Concurrent effects of pregnancy malaria are shown in Fig. 4. The primary events are sequestration of *P*. *falciparum* in the placenta and parasite adherence to gut endothelial cells with altered intestinal barrier function [6, 39]. Adherence of infected erythrocytes containing late developmental stages of the parasite (trophozoites and schizonts) to the endothelium of capillaries and venules, is characteristic of *P. falciparum* infections. Malaria in pregnancy is characterized by the accumulation of infected red blood cells, leukocyte infiltration, and excessive fibrin deposition in the intervillous space of the placenta [40], and is associated with increased risk of low birthweight, attributed to PTB and fetal growth restriction with early development of clinical malaria in infants [41]. Placental intervillositis occurs [36], with induction of oxidative stress biomarkers [42]. Oxidative stress, is an imbalance between oxidants and antioxidants in favour of oxidants, and leads to disruption of redox signalling and physiological function [43]. Placental parasites result in haemozoin formation leading to stimulation of cytokine responses [44, 45], regulation of iNOS expression [46] and NLRP3 inflammasome priming [47]. Haemozoin is an iron-containing pigment which accumulates as cytoplasmic granules in malaria parasites and is a breakdown product of hemoglobin.

The induction of inflammatory cytokines can modulate pregnancy outcomes with beneficial and/or detrimental effects [48]. Maintenance of an appropriate ratio of pro-and anti-inflammatory responses at the feto-maternal interface is a hallmark of successful pregnancy A systemic inflammatory response to malaria during pregnancy leads to increased interleukin (IL)-1β, IL-6, IL-8, IL-10, IL-22, interferon (INF)-Ɣ and soluble tumour necrosis factor (sTNF)-RII in maternal blood [3, 41, 45–51]. Placental IL-6 and IL-8 have been associated with pregnancy loss and PTB [3, 47–49]. These biomarkers are predominantly pro-inflammatory, but some have variable anti-inflammatory effects. Expression of CRP, the synthesis of which is rapidly upregulated with inflammation, occurs principally in hepatocytes, under the control of cytokines originating at the site of pathology [52]. The pro-inflammatory cell signalling upregulates hepcidin [53], with increased iron accumulation in macrophages [16, 37], and reduces iron absorption.

The inflammasome promotes maturation and secretion of pro-inflammatory cytokines IL-1β and IL- 18, which results in pro-inflammatory cell death [54]. Nod-Like Receptor (NLR) P3/P12 dependent activation of caspase-1 is likely to be a key event in mediating systemic production of IL-1β with hypersensitivity to secondary bacterial infection during malaria [55]. Bacterial super-infection is well described in acute malaria, and low-density asymptomatic malaria infections are a risk factor for non-typhi salmonella bacteraemia in children [56] and young adults in Burkina Faso [57]. *Salmonella typhimurium* during malaria may facilitate bacterial colonization [58], and experimental studies in animals indicate shifts in enteric microbiota and increase in susceptibility to intestinal colonization by *S. typhimurium* [59]. In vivo evidence indicates that *S. typhimurium* benefits from iron availability during malaria infection [60]. Higher mean body iron stores, as observed in malaria-positive primigravidae in the PALUFER trial [8], and also reported in a Congolese study [61], may favour colonization with *S. typhimurium.* Evidence is required to substantiate this association in young adults and pregnant women.

### Inflammatory stimuli leading to preterm birth

A maternal cortisol response occurs with inflammation. In non-endemic malaria areas a majority of studies have reported a consistently negative association between maternal cortisol and infant birthweight [62–64]. In contrast studies in malaria endemic areas have reported significant positive associations between cortisol concentration and *P. falciparum* infection prevalence [65, 66]. The process of parturition is associated with inflammation within uterine tissues and it is generally accepted that inflammatory stimuli from multiple extrinsic and intrinsic sources induce labor [67], associated with increases in IL-1β, IL-6 and IL-8 gene expression [68]. Inflammatory stimuli induce labour by affecting progesterone transcriptional activity in uterine cells and causing functional progesterone withdrawal [69]. Progesterone plays a critical role in successful pregnancy and low progesterone concentration is reported in preterm delivery [69], with functional involvement of hormonal and inflammatory stimuli [70, 71], or in experimental animals 24 h following LPS exposure [72]. Hepcidin synthesis in experimental animals has been linked to progesterone receptor membrane component-1 [73]. Young maternal age is important because acquisition of progesterone responsiveness depends on endometrial maturation. Persistence of partial progesterone resistance in adolescents could compromise deep placentation increasing risk of PTB [74].

The characteristics of pregnancy malaria and enteric infection outlined in Fig. 4 indicate a state of chronic inflammation, a maternal cortisol response, progesterone withdrawal and increased risk of PTB. The pathways involved include inflammation-induced cytotoxic effects on progesterone production [75, 76], with progesterone modulation of LPS-induced responses [77, 78]. Metabolic endotoxaemia would impair progesterone production and increase progesterone receptor sensitivity [76, 79, 80]. Concurrent infections including lower genital infections and chorioamnionitis would be expected to be contributory by enhancing activation of the NLRP3 inflammasome, especially with intra-amniotic infection [81]. While the path model is not limited to iron supplemented women, supplementation would influence enteric inflammation before and during pregnancy.

### Path modelling of dual infection, preterm birth and gestation

**Fig. 5:**
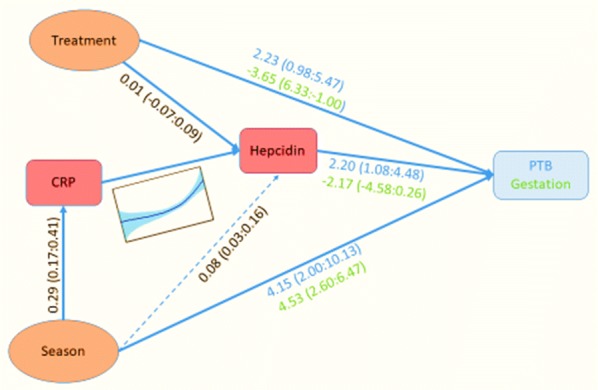
Path model for evaluation of inflammatory factors influencing preterm birth and gestation. See Additional file 3. A fitted path model. Fitted coefficients are shown along the paths and represent effect sizes [differences in log(hepcidin), log(CRP), or gestation, or odds ratios for PTB per unit log(CRP), log(hepcidin)], treatment/non-treatment or amplitude of seasonal variability with 95% CI. The CRP–hepcidin relationship is non-linear and is illustrated graphically (see also Additional file 4: Figure S2). The model fits both PTB and gestation (in days), so there are coefficients for both relationships indicated by blue and green text respective

A path model was constructed based on the schematic in Fig. 4 which incorporated the CRP and hepcidin mediation pathway. Full details are provided in Additional file 4. Figure 5 shows the fitted model along with the estimated coefficients. This model confirmed the seasonal malaria effects on PTB, with mediation through CRP and (non-linear) hepcidin induction. As there was no data on the enteric mediators, this process manifests itself as a strong direct treatment effect on PTB. As in previous analyses, any direct induction of hepcidin from iron supplementation is negligible. An additional effect was detected of season on PTB (broken line in Fig. 5). This may reflect a residual effect due to incomplete measurement of the mediating variables (a single measurement at one time may not capture the full extent of the mediation). Alternatively, it may capture other seasonal effects that are not related to malaria, possibly associated with other infections or nutritional changes.

There was no statistically convincing evidence for any other mechanistic paths beyond the model presented.

## Discussion

The conditions for this PTB model presuppose a setting in which inflammation is due to a common exposure which is experienced by most women, such that the gestational age distribution is shifted to the left with the resultant gestational effect unlikely to be specific to a sub-phenotype [2]. In other words, the inflammatory impact affects most of the population at risk rather than subgroups with selected risk factors. This occurred for women in the PALUFER trial and seems likely to be the case in comparable malaria transmission areas of sub-Saharan Africa where *P. falciparum* prevalence is high, especially in adolescents and primigravidae [82]. A population shift to shorter gestational age has similarly been reported for malaria-exposed primigravidae and secundigravidae in a Tanzanian study [83]. In such settings, the majority of women experience low density, asymptomatic chronic malarial infections that are frequently sub-microscopic and hence remain untreated [84]. With high infection exposure and chronic infections, many primigravidae would experience a sustained inflammatory response spanning the periconceptional period. This is prior to the development of parity-specific malaria immunity which initially develops following malaria infection during the first pregnancy [85].

The inflammatory stimulus defined by CRP was associated with higher hepcidin concentrations with increasing CRP levels in both non-pregnant women and in primigravidae at ANC1. Hepcidin elevation occurred at lower CRP levels (< 5 mg/l) later in gestation at ANC2 (Fig. 3), which was almost sufficient to cancel out the expected late pregnancy physiological suppression of hepcidin [17]. Hepcidin elevation later in gestation is consistent with chronic malaria in pregnancy. Some caution is necessary as numbers are smaller and scattered in post change-point groups, but the analysis is consistent with the hypothesis of hepcidin elevation late in pregnancy in response to malaria and iron-induced gut inflammation. In areas with lower malaria endemicity the inflammatory stimulus would be less, leading to lower hepcidin expression, enhanced gut iron absorption, and less enteric inflammation with reduced risk of PTB. Genital inflammation could additively contribute to hepcidin expression as vaginal lactoferrin, an immune response protein to mucosal infection, was positively associated with serum hepcidin (P = 0.047) in a sub-study using vaginal eluates from these women [15]. If there is a supposition of an inflammatory threshold, this would lead to the timing of human parturition being determined by the trajectory of the inflammatory load increase, and the level of the inflammatory load threshold needed for progesterone signalling [67].

Body iron concentration was higher in women with malaria in this cohort of women [8]. Women with better iron status are more iron replete which would upregulate hepcidin binding to ferroportin, blocking uptake of dietary iron from the intestine. Malaria and gut inflammation in the model also upregulate hepcidin. This has the potential in iron replete women for enhancement of the hepcidin feedback loop leading to an additive or cumulative inflammatory response and increasing risk of PTB. Iron deficient women, who may experience less malaria with correspondingly fewer enteric infections (and possibly fewer genital infections arising from gut contamination) would be at lower risk of entering this cycle with potentially better birth outcomes than iron replete women. In a longitudinal study in Papua New Guinea iron deficiency was associated with substantially reduced odds of low birthweight [86]. The investigators considered the effect was predominantly through malaria independent protective mechanisms, with the association between iron deficiency and PTB restricted to primigravidae, although gestational age was not assessed by ultrasound.

A limitation of this analysis is the scarcity of data for comparative analysis and lack of data on enteric biomarkers or helminthic infections from the PALUFER trial, although all participants received single doses of albendazole and praziquantel at enrolment. Data on serum or red cell folate was not available to assess whether folate status was an additional factor in the model. For example, all women had received 2.8 mg weekly folic acid, a dose which may provide sufficient substrates to enhance folate metabolism in *P. falciparum* infection [87], thereby increasing parasite load and systemic inflammation. Thus, whilst this paper presented and tested a putative causal model, this is not complete and the omission of other pathways and measurement error mean that the results, although suggestive, need to be treated with some caution.

Other field trials assessing periconceptional nutrient interventions have mostly been undertaken in non-malaria endemic areas [14], and effect estimates have prioritized nutritional rather than inflammatory biomarker outcomes. Infection exposures have been poorly defined. In addition, the question arises whether common non-infectious exposures might provide an additional inflammatory stimulus for induction of a hepcidin response sufficient to influence dietary iron absorption. A possible example of this would be prenatal exposure to high levels of ambient air pollution, which has been frequently associated with PTB in non-malaria-endemic areas [88].

### Policy and research implications

Interventions to prevent PTB focus mainly on managing risk factors [89]. Improving the biologic understanding of nutritional and inflammatory mediators, and their seasonal patterns in malaria endemic areas may provide novel ways to identify interventions to reduce inflammatory stimuli. A more clinical perspective requires a focus on female adolescents who face high risk of malaria exposure in their first pregnancy, and in whom inflammation exposure may remain unrecognized due to chronic asymptomatic malaria infection in areas with high malaria transmission. The use of long-term iron supplementation, as recommended by WHO, may be detrimental—even when available on an intermittent seasonal basis, as synergistic enteric inflammatory responses would preclude safety. The occurrence of combined exposures with malaria, enteric, as well as genital infections provides an opportunity to consider other co-infection models [90] and double-hit mechanisms [10], which would necessitate different options for infection control. The role for anti-inflammatory agents, which have been evaluated only for therapeutic use, such as targeting the NLRP3 axis might be considered [91]. However, when malaria and enteric infection co-exist, and if the latter are enhanced by iron supplementation, then this model strongly suggests that malaria control in areas with comparable infection exposures should take precedence to iron supplementation to avoid increasing PTB risk.

## Supplementary information

**Additional file 1.** Background to the PALUFER safety trial of periconceptional iron supplementation. This specifies key published trial results referring to references [2, 5, 7, 8, 13–15].

**Additional file 2.** Laboratory methods. Summary of laboratory methods used for CRP, hepcidin assays and malaria microscopy.

**Additional file 3.** Gestational age distribution in days of livebirths in iron and control arms. Vertical stippled lines indicate 43 weeks and 37 weeks gestation.

**Additional file 4.** Path modelling of PTB and gestation. Outline of Path Model, statistical methodology and results. **Figures S1.** (Path Model); **Figure S2.** (the fitted relationship between CRP and hepcidin); **Table S1.** (Path model coefficients).

## Data Availability

The datasets used and/or analysed during the current study are available from the corresponding author on reasonable request and will be made available following an end user data agreement and sponsor approval.

## Abbreviations

PTB: preterm birth
LPS: lipopolysaccharide
NO: nitric oxide
iNOS: inducible nitric oxide synthase
IL-1β: interleukin 1 beta
IL-6: interleukin 6
IL-8: interleukin 8
IL-10: interleukin 10
IL-18: interleukin 18
IL-22: interleukin 22
INF-Ɣ: interferon gamma
sTNF-RII: soluble Tumour Necrosis Factor Receptor II
TLR: Toll-like receptor
NF-kB: nuclear factor kappa light chain enhancer of activated B cells
NLRP3: Nod-Like Receptor gene coding proteins belonging to the NLRP3 inflammasome complex
CRP: C-reactive protein
ANC1: first study antenatal visit
ANC2: second study antenatal visit
