# Supplementary material for: Testing an infection model to explain excess risk of preterm birth with long-term iron supplementation in a malaria endemic area
Source: Malar J. 2019 Nov 26;18:374. doi: 10.1186/s12936-019-3013-6 (PMC6880560; doi:10.1186/s12936-019-3013-6)
Supplement: Supplementary file 1 — Additional file 1. Background to the PALUFER safety trial of periconceptional iron supplementation. This specifies key published trial results referring to references [2, 5, 7, 8, 13–15]. [file 12936_2019_3013_MOESM1_ESM.docx]

**Additional File 1**

**Background to the PALUFER safety trial of periconceptional iron supplementation**

Between April 2011 and January 2014, a randomized, double blind, controlled trial was conducted amongst nulliparous, non-pregnant residents aged 15–24 years (93% adolescent, < 20 years) in a rural area of Burkina Faso experiencing hyperendemic seasonal malaria [1]. The trial design has been previously described [2]. All participants received a long-lasting insecticidal net and single doses of albendazole (400 mg) and praziquantel at enrolment. Briefly, two cohorts of supplemented women were followed: women remaining non-pregnant and those who experienced pregnancy during, or shortly after, the 18 month iron supplementation period. Nulliparous participants were individually randomized to receive either a weekly capsule containing ferrous gluconate (60 mg) and folic acid (2.8 mg)(n=980), or an identical capsule containing folic acid alone (2.8 mg)(n=979)[3]. A total of 478 women became pregnant. Median weekly supplement adherence up to the first antenatal assessment visit was 79%. A total of 979 women remained non-pregnant and these were assessed for secondary outcomes after 18 months weekly supplementation [3, 4]. The primary study end-point was malaria parasitaemia prevalence at first antenatal visit (ANC1); the secondary end-points were prevalence of anaemia and iron deficiency at ANC1, and the incidence of low birthweight and PTB. Assessment of delivery outcomes provided evidence that long-term iron supplementation lead to excess PTB [5]. This was predominantly associated with the malaria transmission season, with the overall risk of delivery under 37 weeks of 27.5% compared to 13.9% in non-iron-supplemented primigravidae (adjusted P value < 0.001), with a mean gestational age five days shorter (adjusted risk difference, P = 0.012) [4]. In the control arm PTB incidence was consistent with estimates for other known causes such as young age, primiparity and chorioamnionitis in women not receiving periconceptional iron. Weekly iron did not significantly reduce iron deficiency, or anaemia prevalence at ANC1 (mean gestational age 18.5 weeks) [4], which would be consistent with chronic poor iron absorption for individuals living under high malaria pressure. Elevated serum hepcidin was more frequent in parasitemic (22%) compared to non-parasitemic women (11%), (P= 0.015) [3]. *Plasmodium* parasitemia prevalence was 54.3%, at ANC1, and this prevalence did not differ by trial arm [4]. See Additional File 1 for laboratory methods for CRP, hepcidin and malaria microscopy. Free treatment was available for women with fever or other malaria symptoms, but most trial participants were asymptomatic (6.7% with malaria and fever at ANC1). Prevalence of placental malaria parasites at delivery was 33%. In women remaining non-pregnant parasitaemia prevalence was 41% at end assessment after 18 months weekly iron supplementation. Iron-supplemented non-pregnant women also received more antibiotic treatments for non-genital infections (P = 0.014; mainly gastrointestinal infections (P = 0.005), anti-fungal treatments for genital infections (P = 0.014) and analgesics (P = 0.008), than controls [3]. A major strength of this study was its large cohort of non-pregnant peri-menarcheal adolescents (n=1959), as well as a concurrent pregnancy cohort of primigravidae (n= 478). The question arises why excess PTB occurred in women receiving long-term iron supplementation and the possible underlying mechanisms. The Trial’s publications focus on effect estimates for primary and secondary outcomes [3, 4, 5] and nutritional parameters [6, 7], but do not consider the pathophysiological basis for an increased risk of spontaneous PTB. This aspect is addressed in this paper.

Gestational age was estimated by ultrasound examination at ANC1 with a FF Sonic UF-4100 (Fukuda Denshi) scanner. Gestational age was estimated by crown rump length in the first trimester and by bi-parietal diameter, femur length and abdominal circumference afterward.

**References**

1. Rouamba T, Nakanabo-Diallo S, Derra K, Rouamba E, Kazienga A, Inoue Y, et al. Socioeconomic and environmental factors associated with malaria hotspots in the Nanoro demographic surveillance area, Burkina Faso. BMC Public Health 2019;19:249.doi:10.1186/s12889-019-6565-z.
2. Brabin BJ, Gies S, Owens S, Claeys Y, D'Alessandro U, Tinto H, et al.. Perspectives on the design and methodology of periconceptional nutrient supplementation trials. Trials.2016; 17(1):58. doi: 10.1186/s13063-015-1124-0.
3. Brabin L, Roberts SA, Gies S, Nelson A, Diallo S, Stewart CJ, et al. Effects of long-term weekly iron and folic acid supplementation on lower genital tract infection – a double blind, randomised controlled trial in Burkina Faso. BMC Medicine 2017 15:206 DOI 10.1186/s12916-017-0967.
4. Gies S, Diallo S, Roberts SA, Kazienga A, Powney M, Brabin L, et al. Effects of Weekly Iron and Folic Acid Supplements on Malaria Risk in Nulliparous Women in Burkina Faso: A Periconceptional Double-blind Randomized Controlled Non-inferiority Trial. J Infect Dis. 2018. 218(7):1099-109. doi: 10.1093/infdis/jiy257.
5. Brabin B, Gies S, Roberts SA, Diallo S, Lompo OM, Kazienga A, et al. Excess risk of preterm birth with periconceptional iron supplementation in a malaria endemic area: analysis of secondary data on birth outcomes in a double blind randomized controlled safety trial in Burkina Faso. Malar J. 2019;18(1):161. doi: 10.1186/s12936-019-2797-8.
6. Diallo S, Roberts SA, Gies S, Rouamba T, Swinkels DW, Geurts-Moespot AJ, et al. Malaria early in the first pregnancy: potential impact of iron status. Clin Nut 2019; pii: S0261-5614(19)30034-2. [doi.org/](https://doi.org/) 10.1016/j.clnu.2019.01.016.
7. Roberts SA, Brabin L, Diallo S, Gies S, Nelson A, Stewart C, et al. Mucosal lactoferrin response to genital tract infections is associated with iron and nutritional biomarkers in young Burkinabé women^.^ Eur J Clin Nut. 2019;.doi: 10.1038/s41430-019-0444-7.
