# Supplementary material for: Testing an infection model to explain excess risk of preterm birth with long-term iron supplementation in a malaria endemic area
Source: Malar J. 2019 Nov 26;18:374. doi: 10.1186/s12936-019-3013-6 (PMC6880560; doi:10.1186/s12936-019-3013-6)
Supplement: Supplementary file 2 — Additional file 2. Laboratory methods. Summary of laboratory methods used for CRP, hepcidin assays and malaria microscopy. [file 12936_2019_3013_MOESM2_ESM.docx]

**Additional File 2**

**Resume of Laboratory methods**

Blood samples (whole blood and clot activator tubes) were transported within three hours from the field to the central project laboratory at the Clinical Research Unit Nanoro (URCN). Clot activator tubes were centrifuged at 3000 rpm for 10 minutes and sera aliquots stored immediately at minus 80^0^C. C-reactive protein assays were completed within 4-6 months of collection by ELISA (EU59131, IBL International, GMBH, Hamburg, Germany). Ranges for normal controls were 5-8 mg/ml. Serum hepcidin was measured in the Department of Laboratory Medicine (TLM 830), Radboud University Medical Center, Nijmegen, The Netherlands between December 2014 and March 2015 by competitive ELISA assay as previously described [1]. Standards and samples were analyzed in duplicate. Samples giving readings outside the standard curve linear region were repeated at appropriate dilutions. Duplicate readings with coefficient variation >10% were repeated. The lower detection limit of the method was 0.26 nM. The median hepcidin reference level of serum/plasma in a Dutch population was previously determined by Galesloot et al [2]. The hepcidin 95% reference range for women 18-24 yrs age from this Dutch population was: median 2.6nM; 2.5^th^ percentile 0.7nm; and 97.5^th^ percentile 10.5 nM).

Whole blood for malaria films was stained with Giemsa and read independently by two qualified microscopists and data entered in separate files. For discrepant findings (positive/negative; > two-fold difference for parasite densities ≥400/µl; > log10 if < 400/µl), a third independent reading was made, with the mean of the two closest observations accepted as the true value.

1. Kroot JCC, Laarakkers CM, Geurts-Moespot A, Grebenchtchikov N, Pickkers P, van Ede AE, et al. Immunochemical and mass spectrometry-based serum hepcidin assays for a variety of iron metabolism disorders. Clin Chem 2010; 56: 1570-79.
2. Galesloot TE, Vermeulen SH, Geurts-Moespot AJ, Klaver SM, Kroot JJ, van Tienoven D, et al. Serum hepcidin: reference ranges and biochemical correlates in the general population. Blood 2011; 117: e218-25.
