# Supplementary material for: Testing an infection model to explain excess risk of preterm birth with long-term iron supplementation in a malaria endemic area
Source: Malar J. 2019 Nov 26;18:374. doi: 10.1186/s12936-019-3013-6 (PMC6880560; doi:10.1186/s12936-019-3013-6)
Supplement: Supplementary file 3 — Additional file 3. Gestational age distribution in days of livebirths in iron and control arms. Vertical stippled lines indicate 43 weeks and 37 weeks gestation. [file 12936_2019_3013_MOESM3_ESM.docx]

**Additional File 3**

**Fig. Gestational age distribution in days of livebirths in iron and control arms**

Vertical stippled lines indicate 43 weeks and 37 weeks gestation.

Source reference: Brabin B, Gies S, Roberts SA, Diallo S, Lompo OM, Kazienga A, et al. Excess risk of preterm birth with periconceptional iron supplementation in a malaria endemic area: analysis of secondary data on birth outcomes in a double blind randomized controlled safety trial in Burkina Faso. Malar J. 2019;18:161.
