# Supplementary material for: Testing an infection model to explain excess risk of preterm birth with long-term iron supplementation in a malaria endemic area
Source: Malar J. 2019 Nov 26;18:374. doi: 10.1186/s12936-019-3013-6 (PMC6880560; doi:10.1186/s12936-019-3013-6)
Supplement: Supplementary file 4 — Additional file 4. Path modelling of PTB and gestation. Outline of Path Model, statistical methodology and results. Figures S1. (Path Model); Figure S2. (the fitted relationship between CRP and hepcidin); Table S1. (Path model coefficients). [file 12936_2019_3013_MOESM4_ESM.docx]

# **Additional File 4 Path Modelling of PTB and Gestation**

# **3.1 The Path Model (Figure S1)**

# **3.2 Statistical methodology**

# **3.3 Results and Table S1** (Coefficients of the path model) and Figure S2 (Fitted relationship between hepcidin and CRP)

## 3.1 The Path Model

Following the scheme in Figure 4, the PALUFER trial provides data on the two key mediators, CRP and hepcidin, at the late pregnancy ANC2 assessment. Malaria is ubiquitous with the vast majority of women being chronically infected during pregnancy. As infection is highly seasonal and asymptomatic, the time of year over which the pregnancy occurred – as represented by the month of conception - is a good surrogate for the burden of malaria infection. As there is no data available on the mediators in the enteric pathway, these effect are represented by a direct treatment to outcome path. Direct seasonal effects on outcome are included both to capture non-CRP/hepcidin mediated effects and the potential for other seasonal influences beyond those induced by malaria. The pathways are summarised in Figure S1. The fitted model includes all the paths in the schematic of figure 4 for which we have data. Additional paths were considered but not included in the final model and are shown as broken arrows in figure S1.

**Figure S1: The Path Model**


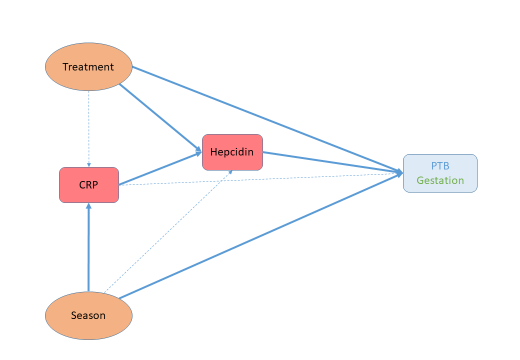


## **3.2 Statistical Methodology**

Models fitted using the brm package [1] and STAN [2] in R [3]. Weakly informative priors were used to aid convergence.

Models are compared using the LOO-IC – a Bayesian generalisation of the usual Akaike Information Criterion based on leave-one-out cross-validation [4]. “Better” fits have a smaller IC. Having fitted the model and estimated the coefficients along each path, the model was refitted omitting each path and estimated the change in LOO-IC based on the posterior distribution. Paths not included in the model were then added in turn to test if omitted paths should be included (LOO-IC decreased by the addition).

A second model was fitted selecting additional paths or excluding paths indicated as improving the fit based on LOO-IC. This model gave almost identical estimates to the one reported so is not presented separately.

Parameters are reported as median and 95% Credible Interval of the posterior distribution – the Bayesian equivalent to the usual mean and 95%CI.

Within this framework we can model both gestation and PTB in a single model. Gestation was modelled as a skewed-normal distribution (significantly left-skewed). Preterm birth (<37w) is modelled as a logistic regression and the estimates are expressed as odds ratios.

C-reactive protein (CRP) and hepcidin were log-transformed. Log(hepcidin) also appeared right-skewed and therefore was fitted using a skewed-normal distribution.

The CRP-hepcidin relationship was expected to be highly non-linear (see Figure 3) so this path was fitted using a smoothing spline to capture the non-linear behaviour, segmented regression being infeasible for this analyses. Season was fitted as a periodic function using a linearised representation based on month of conception: a*sin(2*π*month/12) + b*cos(2*π*month/12) and the parameter reported is the amplitude sqrt(a^2^+b^2^) computed from the posterior distributions of a and b. It is not possible to test for no effect by looking at CI on the amplitude as this is constrained to be positive. All other relationships were fitted as linear as models including non-linear relationships which did not improve the fit.

1. Paul-Christian Buerkner. brms: An R Package for Bayesian Multilevel Models Using Stan. Journal of Statistical Software. 2017; 80(1):1-28. doi:10.18637/jss.v080.i01.
2. https://mc-stan.org
3. R Core Team (2019). R: A language and environment for statistical computing. R Foundation for Statistical Computing, Vienna, Austria. https://www.R-project.org/.
4. Vehtari, A., Gelman, A., and Gabry, J. (2017a). Practical Bayesian model evaluation using leave-one-out cross-validation and WAIC. *Statistics and Computing*. 27(5), 1413–1432. doi:10.1007/s11222-016-9696-4.

**3.3 Results**

Figure 5 summarises the final model. Table S1 shows the fitted values of this model and associated statistics along with the estimated parameters for paths considered, but not included. Figure S2 shows the fitted relationship between hepcidin and CRP.

**Table S1 Coefficients of the path model**

| **Path** | **Estimate** | | **95%CI** | **LOO-IC change if drop** | |  |  |
| --- | --- | --- | --- | --- | --- | --- | --- |
| **Included paths** | | | | | | | |
| Season→log(CRP) | 0.29 | 0.17:0.41 | | 16.0 | |  |  |
| log(CRP)→ log(hepcidin) | (spline: see Figure S2) | | | 23.0 | |  |  |
| Treatment→ log(hepcidin) | 0.01 | -0.07:0.09 | | -2.3 | |  |  |
| log(hepcidin)→PTB (OR) | 2.20 | 1.08:4.48 | | 1.8 | |  |  |
| Treatment→PTB (OR) | 2.23 | 0.98:5.47 | | 0.5 | |  |  |
| Season→PTB (OR) | 4.15 | 2.00:10.13 | | 9.9 | |  |  |
| log(hepcidin)→Gestation (days) | -2.17 | -4.58:0.26 | | 0.4 | |  |  |
| Treatment→Gestation (days) | -3.65 | -6.33:-1.00 | | 6.1 | |  |  |
| Season→Gestation (days) | 4.53 | 2.60:6.48 | | 14.6 | |  |  |
| **Non-included paths** | | | | | **LOO-IC change if add** | |  |
| Treatment→ log(CRP) | 0.02 | -0.15: 0.19 | | 1.3 | |  |  |
| log(CRP)→PTB (OR) | 1.60 | 0.77:3.46 | | 1.0 | |  |  |
| log(CRP)→Gestation (days) | 0.64 | -1.62:2.88 | | 1.3 | |  |  |
| Season→ log(hepcidin) | 0.08 | 0.03:0.16 | | -3.1 | |  |  |

CRP: C-reactive protein; PTB: preterm birth; OR: Odds Ratio; LOO-IC: Leave One Out Cross-validation derived Information Criterion

**Figure S2: Fitted relationship between hepcidin and CRP**.

Shaded area represents the 95%CI.
